# Bioinformatic Analysis of Gene Expression Related to Sialic Acid Biosynthesis in Patients With Medulloblastoma

**DOI:** 10.7759/cureus.59997

**Published:** 2024-05-09

**Authors:** Mudathir Bakhit, Masazumi Fujii

**Affiliations:** 1 Neurosurgery, Fukushima Medical University, Fukushima, JPN

**Keywords:** sialic acid, rna sequence, medulloblastoma, gene expression, bioinformatic analysis

## Abstract

Background

Sialic acid, a critical component for cell membrane integrity, undergoes complex biosynthesis involving enzymes like sialyltransferases (STs), impacting cancer progression. Aberrant sialylation by STs is implicated in cancer growth, invasion, and therapy resistance. Medulloblastoma (MB), a pediatric brain tumor with distinct subgroups and variable genetic alterations, poses uncertainty regarding the implications of sialylation.

Methodology

This study employs bioinformatic analyses on bulk and single-cell RNA-sequenced samples to explore atypical gene expressions linked to sialic acid metabolism in MB. A list of sialic biosynthesis-related genes was compiled using the STRING database. Data of MB samples from bulk and single-cell RNA sequencing were obtained from open-source repositories and were differentially analyzed, focusing on molecular subgroups (WNT, SHH, Group 3, and Group 4). The study employed survival analyses, specifically Cox regression, to analyze the overall survival (OS) data obtained through bulk RNA sequencing.

Results

Thirty-eight genes/proteins related to sialic acid metabolism were identified. Differential expression analysis between WNT and Group 3 and WNT and Group 4 revealed significant differences in seven and eleven genes, respectively, with consistent ST6GAL2 expression disparities (false discovery rate [FDR] *P*-value < 0.01, log2FC > 0.58). Elevated ST6GAL2 expression correlated with improved OS, with mortality risk reductions ranging from 26% to 48% (*P*-value < 0.006, Bonferroni-corrected threshold).

Conclusions

Elevated ST6GAL2 expression correlated with improved OS in diverse MB sample subsets, suggesting potential mechanisms in inhibiting tumor progression and enhancing immune response, requiring experimental validation.

## Introduction

Glycosylation, the attachment of carbohydrates (glycans) to macromolecules like proteins and lipids, is a crucial co-translational and posttranslational modification governing diverse biological functions. Among the various monosaccharides incorporated is the sialic acid or N-acetylneuraminic acids (Neu5Ac), a diverse group of 9-carbon carboxylated monosaccharides synthesized in animals, present at the outermost end of N-linked and O-linked carbohydrate chains and in lipid- and protein-associated glycoconjugates [1].

Sialic acid plays a crucial role in maintaining the integrity and function of the cell membrane. It is synthesized in the cell membrane through a multistep process that involves several enzymatic reactions. The process requires the coordinated work of numerous enzymes that catalyze the biosynthesis, activation, and transfer of sialic acids, leading to the formation of sialyl glycoconjugates. Additionally, the process also involves modifications and degradation of sialyl glycoconjugates and sialic acids [2]. The biosynthesis of sialic acid-containing oligosaccharides and glycoconjugates depends on key enzymes, namely, the sialyltransferases (STs), along with other enzymes like glucosamine (UDP-N-acetyl)-2-epimerase/N-acetylmannosamine kinase (GNE), Neu5Ac 9-phosphate synthase (NANS), Neu5Ac-9-phosphate phosphatase (NANP), and the sialidases (NEU1-4) [2].

Aberrant glycosylation is a critical characteristic of cancerous transformation. Sialic acid and sialoglycans are known to impact tumor progression by promoting the formation of metastases, enabling immune evasion, and contributing to therapy resistance [3,4]. STs, which are expressed differently in cancer cells, are linked to the growth, invasion, and spread of tumors [3-6]. Therefore, targeting the aberrant sialylation process, including enzymes in sialic acid biosynthesis, is a potential cancer treatment strategy [7-10].

Medulloblastoma (MB) is a malignant brain tumor that occurs in children (WHO grade IV). The age of diagnosis usually peaks at around six to eight years old, but it can also occur during infancy or adulthood in some cases. It is an embryonal tumor of the cerebellum and is believed to originate from various neuronal stem or progenitor cell populations during early life [11]. There are four subgroups of MB: Wingless/integrated (WNT), Sonic Hedgehog (SHH), Group 3, and Group 4. Each subgroup is associated with different genetic alterations, age at onset, and prognosis [12,13]. The precise role of aberrant sialylation in MB remains elusive. One study highlighted the substantial cytotoxic efficacy of a poly-guanidine conjugate (GuaDex) in MB cell lines, noting high sialic acid expression in these cells [14]. Yet, the exact implications of hyper- or hyposialylation warrant further exploration. A detailed investigation in this domain could unveil mechanistic insights and therapeutic avenues linked to sialic acid in MB.

In this study, we explore existing databases of MB RNA sequences, employing bioinformatic analyses to identify any atypical gene expressions associated with sialic acid metabolism. This investigation encompasses both bulk tissue and single-cell, RNA-sequenced samples.

## Materials and methods

Search for sialic acid biosynthesis-related genes

We searched for proteins related to sialic acid metabolism using the STRING database (https://string-db.org) [15]. We used the default active interaction sources within the STRING tool, including text mining, experiments, database integration, gene co-expression, neighborhood, gene fusion, and co-occurrence. Text mining involves extracting gene interactions from scientific literature to retrieve comprehensive information from published articles. Experiments involved direct interactions or associations determined through laboratory techniques. Database integration compiled information from diverse biological databases containing experimentally validated or computationally predicted gene interactions. Gene co-expression inferred interactions based on observed correlation patterns in gene expression across conditions or tissues, suggesting potential functional relationships. Neighborhood highlighted genes in close genomic or cellular proximity, implying possible functional associations. Gene fusion identified instances where two separate genes fused, indicating a potential functional association between the originals. Co-occurrence was based on genes frequently occurring together across genomes, indicating possible functional or physical interactions. Moreover, we set the confidence threshold for predicted interactions to 0.7, representing high confidence levels.

Medulloblastoma samples

The study integrated MB samples sourced from two distinctive origins.

Bulk RNA Sequencing (Bulk RNA-seq)

Samples were derived from the International Cancer Genome Consortium (ICGC) PedBrain Tumor Project, encompassing 167 patients [12]. Gene expression analysis relied on data provided by the R2 Genomics Analysis and Visualization Platform (https://hgserver1.amc.nl/) [16].

Single-Cell RNA-seq (scRNA-seq) Sample

**Figure 1:**
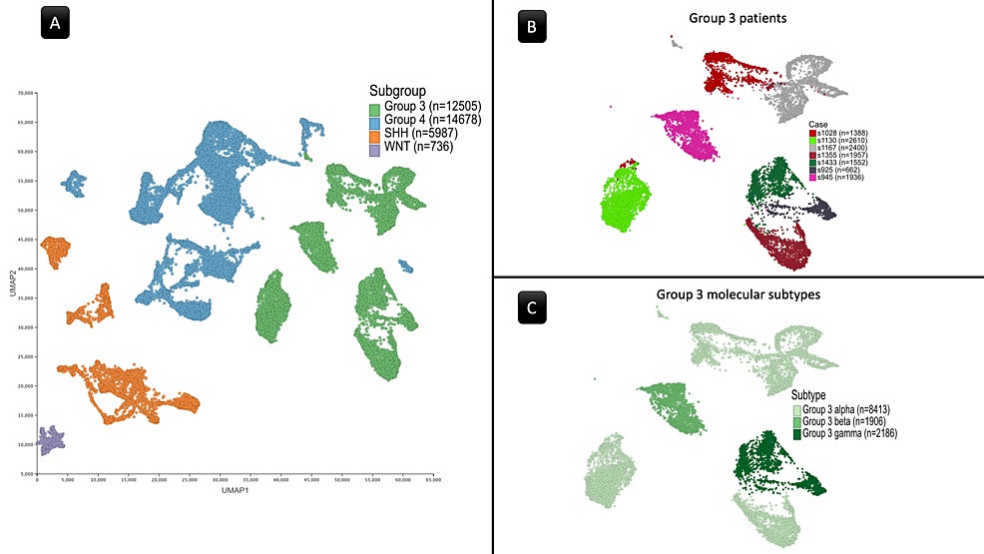
The Uniform Manifold Approximation and Projection (UMAP) of the scRNA-seq sample. (A) UMAP of the entire sample revealing the four principal subgroups of medulloblastoma. (B) UMAP showing patients within the Group 3 subgroup. (C) UMAP highlighting the subtypes within Group 3. Numeric labels indicate the cell count in each cluster.

This dataset focused on childhood MB and comprised 39,946 cells from 28 patients [13]. Gene expression analysis utilized data provided by the R2 Genomics Analysis and Visualization Platform [16]. Additionally, the Uniform Manifold Approximation and Projection (UMAP) technique, accessed through the R2 Genomics Analysis and Visualization Platform and the UCSC Cell Browser (https://www.pneuroonccellatlas.org/; Figure 1), facilitated the visualization and analysis of gene expression profiles and associated data. The scRNA-seq sample represented the primary four subgroups of MB-WNT, SHH, Group 3, and Group 4 (Figure 1A). Further, each subtype underwent subcategorization into molecular subtypes within this cohort. For instance, Figure 1B showcases cases associated with Group 3 MB, while Figure 1C depicts the distribution of α, β, and γ subtypes. Samples with undefined subtypes in the SHH, Group 3, and Group 4 subgroups were excluded from further analyses.

Differential expression analysis

Two-Subgroup Differential Expression Analysis

Employing the R2 Genomics Analysis and Visualization Platform, we conducted a pairwise differential expression analysis comparing the four subgroups within the context of the MB classification (Figure 1A). Notably, the WNT subgroup indicates a favorable survival outcome, contrasting with Groups 3 and 4, which correspond to worse survival [17]. False discovery rate (FDR)-adjusted *P*-values were computed to pinpoint significantly differentially expressed genes between these groups (threshold *P* ≤ 0.01). Additionally, we implemented a fold change threshold of 1.5 (log2FC = 0.58) to identify genes demonstrating substantial expression differences. 

Two-group comparison analyses were conducted using the Mann-Whitney U test, a nonparametric test suitable for non-normally distributed data. The Hodges-Lehmann estimate was calculated as a robust measure of the median difference between paired observations, providing insight into central tendency. Furthermore, the Rank-Biserial Correlation, employed as the effect size for the Mann-Whitney U test, was used to indicate the strength and direction of the relationship between groups. Significant results were visualized using a volcano plot generated in the R statistical package (https://www.r-project.org). Single-cell browsers were used for the visualization of the single-cell expression of the target genes.

Two-Subtype Differential Expression Analysis

Similar to the subgroup analyses, we conducted a pairwise differential expression analysis comparing, when applicable, the molecular subtypes of the scRNA-seq sample (the α and β in WNT; α, β, γ, and δ in SHH; α, β, and γ in Groups 3 and 4) [18].

In our examination of differential gene expression, we incorporated the *MYC *gene into the previously identified list of 38 genes associated with sialic acid biosynthesis. This inclusion aimed to facilitate a comparative analysis between samples characterized by a poorer overall survival (OS), such as Group 3γ, which is characterized by MYC amplification, and those exhibiting a more favorable prognosis, exemplified by the WNT subgroup [12].

Survival analyses 

Utilizing the Bulk RNA-seq dataset, encompassing OS data and events of mortality, we employed the ClinicoPath module within Jamovi, an open-source graphical user interface for the R programming language (https://www.jamovi.org), to perform survival analysis on the target gene expression. The module facilitated Cox regression analyses, enabling the calculation of hazard ratios (HR). Additionally, it assisted in determining a gene expression cut-off, selected by identifying the point where the maximum standardized log-rank statistic is, representing the optimal threshold for dichotomizing patients into high- and low-expression groups. Moreover, the module provided the functionality to assess 1, 3, and 5-year survival rates for these stratified groups. Kaplan-Meier (KM) plots were generated using the log-rank test to visually represent survival probabilities based on the identified gene expression cutoff.

Protein profiling

In our investigation of observed gene/protein expression changes, we utilized the Human Protein Atlas (HPA) database (https://www.proteinatlas.org) [19]. This resource facilitated comparisons between detected alterations and typical gene/protein expression profiles found across diverse human tissues. Leveraging this approach, we contextualized and interpreted the identified changes within the spectrum of typical gene/protein expression patterns observed in healthy tissues relative to MB origin.

The RNA HPA brain gene data illustrate transcript expression levels summarized per gene across 193 subregions using RNA-seq analysis. Our imported dataset includes crucial information: Ensembl gene identifiers ('Gene'), analyzed samples labeled by their respective 'Subregions,' transcripts per million ('TPM'), protein-transcripts per million ('pTPM'), and normalized expression ('nTPM'). To augment our analysis, we apply a log2 transformation to the 'nTPM' values, expressing these transformed values as mean ± standard deviation for each major region relative to the MB origin (cerebellum and brain stem). Providing detailed breakdowns of subregions in separate charts enables a comprehensive representation of the observed values. Moreover, our approach extends to a meticulous review of the data related to antibody staining. This thorough examination significantly contributes to enhancing our understanding of the observed protein expression patterns.

## Results

List of target genes/proteins

**Figure 2:**
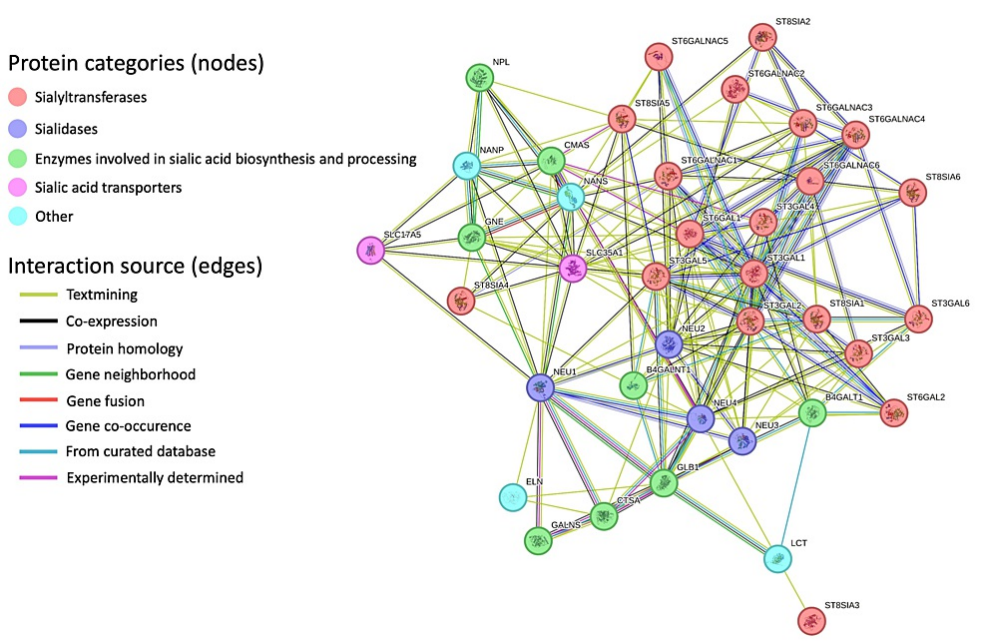
STRING tool protein-protein interaction network related to sialic acid metabolism. The tool presented the interactions between 38 proteins. Network nodes represent proteins, where each node represents all the proteins produced by a single protein-coding gene locus. Edges represent protein-protein associations. Different edge colors represent different kinds of evidence of interaction between the proteins.

The STRING tool search found 38 genes/proteins related to sialic acid metabolism (Figure 2). They can be grouped into five groups:

(1) Sialyltransferases (STs): ST3GAL1, ST3GAL2, ST3GAL3, ST3GAL4, ST3GAL5, ST3GAL6, ST6GAL1, ST6GAL2, ST6GALNAC1, ST6GALNAC2, ST6GALNAC3, ST6GALNAC4, ST6GALNAC5, ST6GALNAC6, ST8SIA1, ST8SIA2, ST8SIA3, ST8SIA4, ST8SIA5, ST8SIA6.

(2) Sialidases: NEU1, NEU2, NEU3, NEU4.

(3) Enzymes involved in sialic acid biosynthesis and processing: B4GALNT1, B4GALT1, CMAS, CTSA, GALNS, GLB1, GNE, NPL.

(4) Sialic acid transporters: SLC17A5, SLC35A1.

(5) Other associated with sialic acid metabolism: NANP, NANS, LCT, ELN.

Differential expression analysis

**Figure 3:**
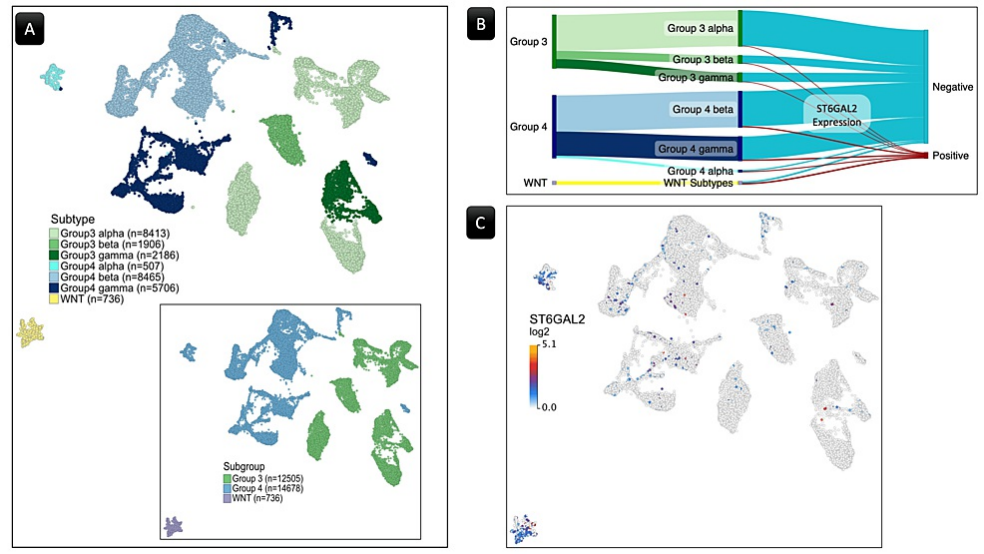
The ST6GAL2 gene expression. (A) A UMAP plot displays the spatial distribution of cells representing WNT, Group 3, and Group 4 medulloblastoma subgroups and their subtypes. Each dot represents a single cell, with distinct clusters representing the three subgroups, highlighting their spatial arrangement. The SHH was excluded since it lacked significant relevance in the current work. (B) A Sankey flow diagram illustrating the distribution of samples expressing ST6GAL2 across medulloblastoma subgroups and subtypes. (C) The UMAP plot illustrates the log2-transformed expression levels of the ST6GAL2 gene across the WNT, Group 3, and Group 4  subgroups. WNT, wingless/integrated; UMAP, Uniform Manifold Approximation and Projection; ST6GAL2, α-2,6-sialyltransferase 2

After excluding cells lacking defined subtypes, the dataset obtained from the R2 Genomics Analysis and Visualization Platform contained 12,505 cells in Group 3, 14,678 cells in Group 4, 5,987 cells in the SHH subgroup, and 736 cells in the WNT subgroup. Since WNT lacked subtype classification, it was treated as a single subtype (Figure 3A). Regarding the SHH subgroup, no beta or gamma subtypes were available. Furthermore, in the analysis of differential gene expression, no genes showed a significant result when applying the SHH subgroup or its available subtypes. According to this and given the greater relevance of survival data from WNT and Groups 3&4 to the study, the SHH sample was excluded from further analysis or discussion beyond the differential gene expression analysis.

**Figure 4:**
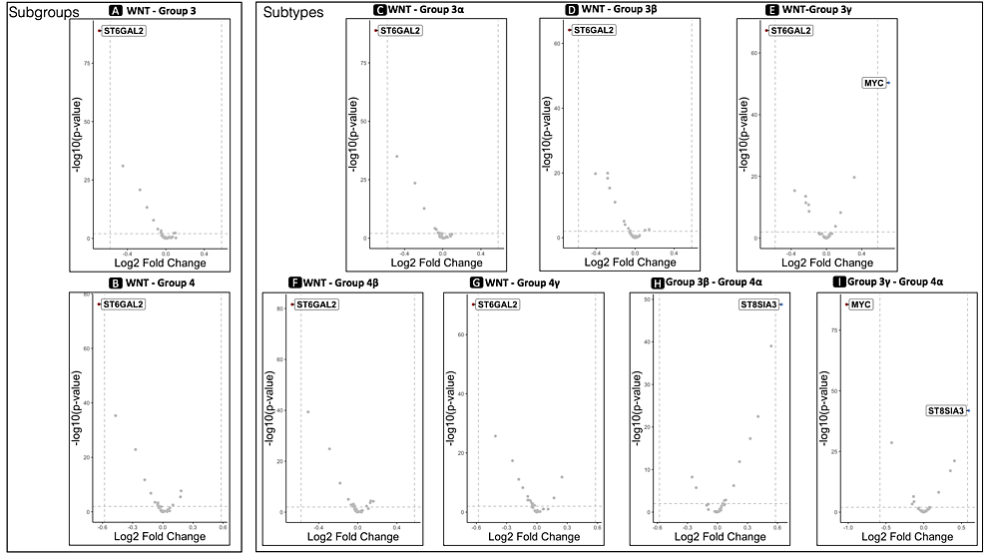
Differential expression analysis. Volcano plots illustrate the ST6GAL2 differential gene expression between wingless/integrated (WNT) and subgroups Groups 3 (A) and 4 (B), along with their subtypes 3α (C), 3β (D), 3γ (E), 4β (F), and 4γ (G). Also, the ST8SAI3 differential gene expression between Group 4α and subtypes Group 3β (H) and 3γ (I). In the plot, genes related to sialic acid metabolism are highlighted only when showing a fold change exceeding the predefined threshold (log2FC = 0.69), signifying significant expression differences compared to other genes. Specifically, ST6GAL2 was highlighted in all tests comparing WNT to Groups 3 and 4, including their subtypes, showing higher expression in the latter. ST8SIA3 was highlighted in the differential expression analyses between subtype Group 4α and Group 3β and γ, showing higher expression in the former. Additionally, the MYC gene, once exhibiting a fold change higher than the predefined threshold, is also emphasized. Positive values of log2FC indicate higher expression in the group mentioned first in the title, while negative values suggest higher expression in the second group. ST6GAL2, α-2,6-sialyltransferase 2

The analysis of differential gene expression between the WNT and Group 3 revealed seven genes displaying a significant FDR *P*-value of ≤ 0.01 (Table 1). Six out of the seven belong to the STs. Notably, α-2,6-sialyltransferase 2 (ST6GAL2), a member of the STs and the second member of the ST6GAL was the only gene that showed significant expressional differences between the two groups, with a log2FC of 0.69 (Figure 4A).

**Table 1:** Results of the two-group differential expression analysis in the scRNA-seq sample-subgroups. Only results with an FDR-corrected *P*-value ≤ 0.01 are shown. Results in bold font have a fold change ≥ 1.5 (log2FC ≥ 0.58). SHH, Sonic Hedgehog; WNT, wingless/integrated; FDR, false discovery rate; ST6GAL2, α-2,6-sialyltransferase 2

Comparison	Gene	*P*-value	FDR-corrected *P*-value	log2-fold change
WNT-SHH	ST3GAL1	1.80E-19	3.41E-18	-0.27
	ST8SIA1	2.91E-15	3.68E-14	0.2
	ST3GAL5	1.16E-14	1.10E-13	-0.23
	ST6GAL2	1.11E-09	7.02E-09	-0.23
	ST6GAL1	1.08E-09	8.18E-09	0.16
	ELN	3.02E-08	1.64E-07	-0.25
	B4GALNT1	2.73E-05	1.30E-04	0.1
	ST8SIA3	5.37E-04	2.27E-03	0.13
WNT-Group 3	ST6GAL2	1.30E-89	4.93E-88	-0.69
	ELN	9.84E-32	1.87E-30	-0.45
	ST3GAL1	1.64E-21	2.08E-20	-0.27
	ST8SIA4	5.39E-14	5.13E-13	-0.2
	ST8SIA3	1.67E-08	1.27E-07	-0.13
	ST3GAL2	1.04E-04	6.58E-04	-0.08
	ST3GAL5	5.46E-04	2.97E-03	-0.05
WNT-Group 4	ST6GAL2	6.15E-77	2.27E-75	-0.64
	ELN	4.89E-36	9.04E-35	-0.47
	ST8SIA4	1.80E-12	1.67E-11	-0.18
	ST6GAL1	1.99E-08	1.47E-07	0.18
	ST3GAL5	1.44E-07	8.89E-07	-0.12
	ST8SIA2	3.14E-06	1.66E-05	0.18
	ST3GAL4	2.98E-04	1.38E-03	-0.08
	ST3GAL1	4.68E-04	1.92E-03	-0.05
	ST3GAL2	1.25E-03	4.63E-03	-0.05
	GALNS	1.74E-03	5.85E-03	-0.05
	ST3GAL6	2.99E-03	9.23E-03	0.1
SHH-Group 3	ST6GAL2	2.04E-283	7.74E-282	-0.46
	ST8SIA3	1.76E-104	2.22E-103	-0.26
	ST8SIA1	1.05E-60	9.93E-60	-0.16
	ELN	5.74E-54	4.36E-53	-0.19
	ST8SIA4	1.51E-34	9.58E-34	-0.12
	B4GALNT1	1.32E-33	7.16E-33	-0.12
	ST3GAL5	1.60E-21	7.59E-21	0.18
	ST8SIA5	3.92E-21	1.66E-20	-0.09
	ST6GAL1	5.36E-17	2.04E-16	-0.07
	ST8SIA2	1.25E-15	4.33E-15	0.11
	GALNS	6.95E-08	2.20E-07	-0.04
	CTSA	1.64E-07	4.81E-07	-0.02
	NANS	1.93E-07	5.24E-07	0.11
	CMAS	5.27E-07	1.33E-06	-0.01
	GNE	2.35E-06	5.58E-06	-0.03
	ST3GAL6	6.45E-06	1.44E-05	-0.04
	ST3GAL2	2.26E-05	4.77E-05	-0.02
	ST3GAL4	4.05E-05	8.10E-05	-0.02
	SLC35A1	4.72E-05	8.97E-05	-0.02
	ST6GALNAC4	8.56E-05	1.55E-04	-0.03
	NEU1	9.57E-04	1.65E-03	0.07
	ST6GALNAC3	2.17E-03	3.59E-03	-0.03
	GLB1	3.15E-03	4.98E-03	-0.01
	ST3GAL3	5.53E-03	8.41E-03	0.01
SHH-Group 4	ST6GAL2	2.56E-236	9.72E-235	-0.4
	ELN	1.57E-71	2.99E-70	-0.22
	ST8SIA1	8.60E-59	1.09E-57	-0.14
	ST8SIA3	9.30E-47	8.83E-46	-0.14
	ST3GAL1	8.71E-39	6.62E-38	0.22
	ST8SIA2	8.00E-37	5.07E-36	0.19
	ST8SIA4	9.06E-30	4.92E-29	-0.11
	ST3GAL4	8.06E-20	3.83E-19	-0.07
	ST8SIA5	1.35E-17	5.71E-17	-0.08
	GALNS	1.58E-09	5.99E-09	-0.04
	ST3GAL5	1.04E-08	3.59E-08	0.11
	B4GALNT1	1.45E-07	4.59E-07	-0.03
	GNE	4.97E-07	1.45E-06	-0.03
	CMAS	6.43E-07	1.75E-06	0
	ST6GALNAC4	9.09E-07	2.30E-06	-0.04
	ST3GAL3	3.64E-05	8.66E-05	0.01
	CTSA	8.92E-05	1.99E-04	0
	NANS	1.57E-04	3.31E-04	0.09
	ST6GALNAC3	4.32E-04	8.65E-04	-0.03
	GLB1	1.64E-03	2.96E-03	-0.01
	SLC35A1	2.31E-03	3.98E-03	0
	ST3GAL2	5.26E-03	8.70E-03	0.01
Group 3-Group 4	ST3GAL1	2.77E-67	5.25E-66	0.22
	ST8SIA3	3.21E-20	4.06E-19	0.11
	ST6GAL1	2.18E-19	2.07E-18	0.1
	B4GALNT1	4.81E-18	3.66E-17	0.09
	ST3GAL4	1.82E-09	1.15E-08	-0.05
	NEU1	5.63E-09	3.06E-08	-0.05
	ST8SIA2	7.64E-09	3.63E-08	0.08
	ST3GAL6	2.72E-08	1.15E-07	0.06
	ST3GAL5	5.34E-07	2.03E-06	-0.07
	ST6GAL2	2.90E-06	1.00E-05	0.06
	SLC17A5	6.26E-04	1.98E-03	-0.03

The analysis of differential gene expression between the WNT and Group 4 showed eleven genes displaying a significant FDR p-value of ≤ 0.01 (Table 1). Nine belong to the STs. Again, ST6GAL2 was the only gene that showed significant expressional differences between the two groups, with a log2FC of 0.64 (Figure 4B). No other pairwise analyses within the four subgroups yielded statistically significant results in terms of gene expression alterations.

Despite the notable discrepancy in cell counts between the subgroups, with 736 cells in WNT, 12,505 in Group 3, and 14,678 in Group 4, the ratio of cells exhibiting ST6GAL2 expression was substantially higher in the WNT subgroup (Figure 3B and Table 2). Figure 3C depicts the cells with ST6GAL2 log2-transformed expression values in the three groups, highlighting the higher ratio of the cells within the WNT cluster.

**Table 2:** The contingency table of the frequency of ST6GAL2 expression pattern within the WNT, Group 3, and Group 4 subgroups of the medulloblastoma. ST6GAL2, α-2,6-sialyltransferase 2; WNT, wingless/integrated

Subgroup		ST6GAL2 status		Total
		Expressed	Not expressed	
Group 4	Cell count	785	13,893	14,678
	% within subgroup	5%	95%	100%
Group 3	Cell count	261	12,244	12,505
	% within subgroup	2%	98%	100%
WNT	Cell count	337	399	736
	% within subgroup	46%	54%	100%

Moreover, an investigation into the pairwise differential expression of the 38 genes among the subtypes unveiled statistically significant variances (Table 3). Specifically, ST6GAL2 exhibited notable differences in expression levels between the WNT subtype and Groups 3 (with log2FC values of 0.70, 0.67, and 0.68 compared to subtypes 3α, β, and γ, respectively, shown in Figures 4C-4E) as well as Group 4, excluding Group 4α (with log2FC values of 0.67 and 0.63 compared to Groups 4β and γ, respectively, shown in Figures 4F-4G).

**Table 3:** Results of the two-group differential expression analysis in the scRNA-seq sample-subtypes. Only results with an FDR-corrected *P*-value ≤ 0.01 are shown. Results in bold font have a fold change ≥ 1.5 (log2FC ≥ 0.58). FDR, false discovery rate; WNT, wingless/integrated; SHH, Sonic Hedgehog

Comparison	Gene	FDR-corrected *P*-value	log2-fold change
WNT-SHHα	ELN	8.62E-12	-0.31
	ST3GAL1	3.16E-18	-0.27
	ST3GAL5	1.42E-17	-0.26
	ST6GAL2	4.05E-08	-0.22
	ST8SIA4	9.15E-03	-0.08
	ST3GAL6	2.73E-03	0.09
	B4GALNT1	1.22E-05	0.12
	ST8SIA3	4.18E-03	0.13
	ST8SIA1	2.95E-11	0.18
	ST6GAL1	1.39E-10	0.18
WNT-SHHδ	ST6GAL2	2.84E-08	-0.3
	ST3GAL1	4.40E-11	-0.26
	CMAS	9.50E-04	0.08
	ST3GAL3	1.05E-04	0.11
	ST8SIA3	2.79E-03	0.11
	ST8SIA5	1.93E-13	0.21
	ST8SIA1	7.96E-21	0.29
WNT-Group 3α	ST6GAL2	5.27E-88	-0.7
	ELN	2.00E-34	-0.48
	ST3GAL1	3.55E-23	-0.29
	ST8SIA4	1.92E-12	-0.19
	ST3GAL2	5.17E-04	-0.08
	ST8SIA3	1.37E-03	-0.07
WNT-Group 3β	ST6GAL2	2.63E-63	-0.67
	ELN	1.92E-19	-0.41
	ST3GAL5	3.73E-18	-0.28
	ST8SIA3	1.98E-19	-0.28
	ST3GAL1	3.19E-15	-0.26
	ST8SIA4	5.98E-11	-0.21
	ST3GAL2	3.96E-05	-0.11
	GALNS	5.10E-03	-0.07
	NANS	9.42E-03	0.14
WNT-Group 3γ	ST6GAL2	1.94E-66	-0.68
	ELN	3.61E-15	-0.36
	ST8SIA3	1.95E-13	-0.24
	ST3GAL5	2.02E-11	-0.23
	ST8SIA4	7.58E-11	-0.2
	ST3GAL1	9.29E-09	-0.2
	ST8SIA2	5.18E-04	0.1
	ST6GAL1	2.08E-08	0.16
	NANS	2.32E-19	0.31
WNT-Group 4α	ELN	9.72E-18	-0.51
	ST3GAL5	3.49E-08	-0.26
	ST3GAL1	9.12E-05	-0.19
	ST8SIA4	7.66E-05	-0.17
	ST3GAL2	5.10E-03	-0.14
	NANS	6.38E-03	-0.11
	ST3GAL4	9.87E-03	-0.1
	ST8SIA1	5.37E-03	0.12
	B4GALNT1	2.33E-05	0.17
	ST6GAL1	6.78E-10	0.24
	CMAS	3.26E-08	0.28
	ST8SIA3	2.05E-10	0.36
	ST8SIA2	4.91E-25	0.51
WNT-Group 4β	ST6GAL2	8.60E-81	-0.67
	ELN	7.50E-39	-0.51
	ST8SIA4	3.59E-11	-0.18
	ST3GAL5	6.44E-05	-0.1
	ST3GAL4	4.72E-03	-0.07
	ST3GAL1	2.68E-03	-0.04
	ST3GAL6	9.00E-04	0.13
	ST6GAL1	2.19E-04	0.13
	ST8SIA2	2.91E-04	0.16
WNT-Group 4γ	ST6GAL2	2.32E-69	-0.63
	ELN	3.77E-25	-0.41
	ST8SIA4	6.04E-11	-0.18
	ST3GAL5	3.39E-08	-0.14
	ST3GAL4	3.16E-04	-0.09
	CMAS	2.66E-05	-0.09
	NEU1	3.44E-04	-0.08
	ST3GAL2	5.42E-04	-0.06
	GALNS	2.80E-03	-0.05
	NANS	7.93E-03	-0.05
	ST3GAL1	4.07E-03	-0.04
	ST8SIA2	7.39E-05	0.17
	ST6GAL1	1.36E-11	0.25
SHH α-SHH δ	ST6GAL1	2.22E-05	-0.12
	B4GALNT1	4.49E-04	-0.1
	ST3GAL6	5.94E-03	-0.07
	NEU1	6.41E-03	0.04
	ST8SIA4	2.66E-04	0.06
	ST3GAL3	2.74E-04	0.06
	CTSA	4.36E-04	0.07
	ST3GAL4	1.68E-06	0.08
	ST8SIA1	2.07E-07	0.11
	ST8SIA2	8.42E-07	0.12
	CMAS	3.99E-13	0.14
	ST3GAL5	9.16E-16	0.17
	ST8SIA5	2.76E-20	0.18
	ELN	1.82E-29	0.34
SHH α-Group 3α	ST6GAL2	1.14E-224	-0.48
	ST8SIA3	1.70E-47	-0.2
	ELN	2.56E-31	-0.17
	B4GALNT1	2.57E-34	-0.15
	ST8SIA1	3.88E-36	-0.14
	ST8SIA4	1.81E-19	-0.11
	ST6GAL1	8.45E-22	-0.1
	ST8SIA5	1.60E-06	-0.06
	ST3GAL6	7.07E-06	-0.04
	GALNS	6.32E-05	-0.04
	GNE	5.98E-05	-0.03
	ST6GALNAC4	4.51E-03	-0.03
	ST3GAL4	5.35E-05	-0.02
	ST3GAL2	8.88E-04	-0.02
	SLC35A1	9.25E-04	-0.02
	CTSA	6.62E-03	0
	ST8SIA2	5.37E-14	0.13
	ST3GAL5	2.99E-56	0.31
SHH α-Group 3β	ST6GAL2	1.84E-87	-0.45
	ST8SIA3	1.36E-81	-0.41
	ST6GAL1	9.56E-19	-0.16
	ST8SIA1	9.26E-15	-0.13
	ST8SIA4	2.66E-10	-0.12
	B4GALNT1	6.93E-10	-0.11
	ELN	3.83E-06	-0.09
	ST8SIA5	1.93E-03	-0.06
	GALNS	3.33E-04	-0.06
	SLC35A1	3.48E-04	-0.05
	ST3GAL2	3.48E-04	-0.05
	ST6GALNAC4	4.62E-03	-0.05
	GNE	3.33E-04	-0.05
	ST3GAL6	1.62E-03	-0.05
	NEU3	9.12E-03	-0.04
	ST3GAL3	1.71E-03	-0.03
	ST8SIA2	7.18E-10	0.14
	NANS	1.94E-12	0.19
SHH α-Group 3γ	ST6GAL2	1.96E-97	-0.46
	ST8SIA3	6.85E-66	-0.37
	B4GALNT1	3.05E-15	-0.14
	ST8SIA1	1.33E-16	-0.14
	ST8SIA4	1.81E-10	-0.12
	ST3GAL6	1.89E-04	-0.06
	ST8SIA5	7.73E-04	-0.06
	CTSA	1.95E-03	-0.05
	ST3GAL4	6.63E-05	0.06
	ST3GAL1	2.76E-04	0.08
	NEU1	8.50E-14	0.12
	ST8SIA2	7.05E-16	0.14
	NANS	4.35E-71	0.36
SHH α-Group 4α	ELN	1.13E-06	-0.2
	ST8SIA3	5.24E-07	0.23
	CMAS	7.13E-17	0.33
	ST8SIA2	3.14E-44	0.55
SHH α-Group 4β	ST6GAL2	1.34E-199	-0.45
	ELN	1.99E-40	-0.19
	ST8SIA3	4.00E-37	-0.16
	ST8SIA1	1.41E-36	-0.14
	ST8SIA4	4.55E-16	-0.1
	ST8SIA5	2.51E-05	-0.05
	ST6GAL1	8.82E-08	-0.04
	ST3GAL4	3.46E-07	-0.04
	GNE	1.14E-05	-0.04
	GALNS	5.42E-05	-0.04
	B4GALNT1	1.18E-04	-0.03
	ST6GALNAC4	7.11E-03	-0.03
	ST3GAL3	2.08E-04	-0.01
	NANS	2.67E-15	0.16
	ST3GAL5	4.64E-19	0.17
	ST8SIA2	7.47E-29	0.2
	ST3GAL1	4.96E-30	0.23
SHH α-Group 4γ	ST6GAL2	1.06E-144	-0.41
	ST8SIA3	1.48E-30	-0.15
	ST8SIA1	1.47E-24	-0.11
	ELN	9.09E-14	-0.1
	ST8SIA4	5.95E-14	-0.1
	B4GALNT1	3.68E-16	-0.09
	ST3GAL4	3.50E-10	-0.07
	ST6GALNAC4	1.04E-04	-0.05
	GALNS	2.39E-06	-0.04
	ST8SIA5	1.16E-03	-0.04
	CMAS	4.46E-09	-0.03
	ST3GAL6	1.12E-03	-0.03
	GNE	2.29E-04	-0.02
	CTSA	4.45E-04	-0.01
	SLC35A1	3.80E-03	0
	ST3GAL2	3.78E-03	0
	ST3GAL5	3.46E-07	0.12
	ST8SIA2	7.00E-28	0.21
	ST3GAL1	8.28E-26	0.23
SHH δ-Group 3α	ELN	1.46E-73	-0.5
	ST6GAL2	6.98E-70	-0.4
	ST8SIA1	1.17E-36	-0.25
	ST8SIA5	3.76E-36	-0.24
	ST8SIA3	1.02E-19	-0.18
	ST8SIA4	2.88E-20	-0.17
	CMAS	1.75E-16	-0.11
	ST3GAL4	1.16E-13	-0.1
	CTSA	1.64E-07	-0.08
	GALNS	5.63E-04	-0.05
	ST6GALNAC3	8.24E-03	-0.05
	B4GALNT1	6.94E-03	-0.04
	ST6GALNAC4	5.76E-04	-0.04
	ST3GAL3	3.27E-05	-0.03
	ST3GAL2	6.75E-04	-0.03
SHH δ-Group 3β	ELN	1.91E-40	-0.43
	ST8SIA3	7.17E-49	-0.4
	ST6GAL2	2.95E-44	-0.37
	ST8SIA1	1.78E-25	-0.24
	ST8SIA5	2.89E-26	-0.24
	ST3GAL5	1.28E-16	-0.19
	ST8SIA4	3.06E-16	-0.18
	CMAS	3.81E-14	-0.13
	ST3GAL3	1.58E-08	-0.09
	CTSA	3.18E-06	-0.08
	GALNS	1.67E-04	-0.07
	ST3GAL4	5.20E-06	-0.06
	ST3GAL2	6.74E-05	-0.06
	ST6GALNAC4	2.01E-04	-0.06
	SLC35A1	1.05E-03	-0.05
	GNE	6.60E-03	-0.03
	NANS	5.03E-06	0.21
SHH δ-Group 3γ	ELN	1.23E-33	-0.38
	ST6GAL2	1.02E-46	-0.38
	ST8SIA3	1.19E-38	-0.35
	ST8SIA1	2.28E-27	-0.25
	ST8SIA5	3.23E-28	-0.24
	ST8SIA4	3.16E-16	-0.18
	CMAS	6.84E-13	-0.15
	ST3GAL5	4.28E-09	-0.14
	CTSA	2.27E-08	-0.13
	ST3GAL3	5.95E-03	-0.05
	ST6GAL1	6.80E-04	0.11
	NANS	9.46E-34	0.38
SHH δ-Group 4α	ELN	2.16E-30	-0.54
	ST8SIA5	9.95E-11	-0.21
	ST8SIA1	1.09E-06	-0.17
	ST3GAL5	3.21E-06	-0.17
	ST3GAL4	4.22E-08	-0.15
	ST8SIA4	3.59E-06	-0.15
	B4GALNT1	3.69E-04	0.16
	ST6GAL2	4.12E-03	0.17
	ST6GAL1	3.03E-06	0.18
	CMAS	1.18E-03	0.2
	ST8SIA3	2.79E-05	0.24
	ST8SIA2	2.26E-19	0.43
SHH δ-Group 4β	ELN	1.27E-81	-0.53
	ST6GAL2	1.20E-61	-0.37
	ST8SIA1	6.72E-37	-0.25
	ST8SIA5	2.48E-34	-0.23
	ST8SIA4	3.41E-18	-0.16
	ST8SIA3	5.80E-16	-0.15
	ST3GAL4	8.23E-16	-0.12
	CMAS	7.93E-13	-0.09
	ST3GAL3	1.86E-09	-0.08
	ST6GALNAC3	4.37E-03	-0.05
	GALNS	4.45E-04	-0.05
	CTSA	4.14E-05	-0.04
	ST6GALNAC4	6.49E-04	-0.03
	ST3GAL5	3.78E-03	-0.01
	ST3GAL6	1.20E-03	0.11
	NANS	2.27E-04	0.17
	ST3GAL1	1.12E-07	0.22
SHH δ-Group 4γ	ELN	6.22E-55	-0.44
	ST6GAL2	1.49E-49	-0.33
	ST8SIA1	1.65E-30	-0.22
	ST8SIA5	2.53E-30	-0.22
	CMAS	2.15E-26	-0.17
	ST8SIA4	1.37E-17	-0.16
	ST3GAL4	1.20E-18	-0.14
	ST8SIA3	8.00E-15	-0.14
	CTSA	1.35E-08	-0.08
	ST6GALNAC3	4.18E-03	-0.05
	GALNS	5.01E-05	-0.05
	ST6GALNAC4	2.78E-05	-0.05
	ST3GAL5	3.21E-06	-0.05
	NEU1	1.01E-05	-0.04
	ST3GAL2	1.28E-03	-0.01
	ST3GAL3	2.37E-04	0
	ST6GAL1	7.68E-07	0.19
	ST3GAL1	2.02E-07	0.22
Group 3α-Group 3β	ST3GAL5	1.19E-35	-0.33
	ST8SIA3	5.57E-21	-0.21
	NANS	2.00E-17	0.18
Group 3α-Group 3γ	ST3GAL5	4.39E-22	-0.28
	ST8SIA3	4.04E-11	-0.17
	ST3GAL2	5.35E-03	0.02
	SLC17A5	8.23E-03	0.02
	NEU1	2.07E-09	0.06
	ST3GAL4	1.04E-13	0.08
	ST6GAL1	2.10E-10	0.09
	ST3GAL1	3.69E-08	0.09
	ELN	3.30E-08	0.12
	NANS	5.44E-86	0.35
Group 3α-Group 4α	ST3GAL5	9.97E-09	-0.31
	ST3GAL1	3.25E-03	0.1
	ST6GAL1	3.75E-09	0.16
	B4GALNT1	4.81E-10	0.2
	CMAS	3.22E-18	0.31
	ST8SIA2	6.72E-28	0.41
	ST8SIA3	3.62E-26	0.43
	ST6GAL2	1.54E-50	0.57
Group 3α-Group 4β	ST3GAL5	1.79E-15	-0.15
	ST3GAL3	2.76E-03	-0.04
	ST3GAL2	3.93E-03	0.04
	ST6GAL1	1.31E-05	0.06
	ST8SIA2	1.26E-04	0.06
	ST3GAL6	6.17E-09	0.09
	B4GALNT1	5.96E-20	0.12
	NANS	2.62E-28	0.14
	ST3GAL1	1.93E-54	0.25
Group 3α-Group 4γ	ST3GAL5	1.16E-24	-0.19
	NEU1	2.57E-06	-0.07
	CMAS	7.82E-05	-0.06
	B4GALNT1	4.81E-03	0.06
	ELN	2.49E-03	0.07
	ST6GAL2	2.06E-04	0.07
	ST8SIA2	2.82E-05	0.07
	ST6GAL1	5.34E-28	0.17
	ST3GAL1	6.67E-44	0.25
Group 3β-Group 3γ	ST3GAL3	1.84E-03	0.04
	NEU1	2.05E-04	0.04
	ST3GAL4	8.67E-04	0.04
	SLC35A1	4.19E-03	0.05
	ST3GAL2	1.06E-03	0.05
	ST3GAL5	4.78E-03	0.05
	SLC17A5	1.55E-03	0.06
	ST3GAL1	4.55E-03	0.06
	ST6GAL1	2.87E-12	0.14
	NANS	1.12E-13	0.17
Group 3β-Group 4α	NANS	3.29E-08	-0.25
	GNE	6.54E-03	0.07
	SLC35A1	5.44E-03	0.08
	B4GALNT1	2.96E-06	0.16
	ST6GAL1	9.79E-12	0.22
	CMAS	4.52E-17	0.33
	ST8SIA2	3.82E-22	0.41
	ST6GAL2	1.65E-38	0.54
	ST8SIA3	5.48E-48	0.64
Group 3β-Group 4β	ELN	1.19E-04	-0.1
	ST3GAL4	4.27E-03	-0.06
	ST3GAL2	1.75E-03	0.07
	B4GALNT1	6.03E-04	0.08
	ST3GAL6	3.59E-04	0.09
	ST6GAL1	2.42E-07	0.12
	ST3GAL5	7.54E-15	0.18
	ST3GAL1	5.88E-15	0.22
	ST8SIA3	5.29E-26	0.25
Group 3β-Group 4γ	NANS	1.69E-17	-0.19
	NEU1	3.10E-04	-0.09
	ST3GAL4	1.25E-04	-0.08
	ST3GAL3	7.35E-03	0.09
	ST3GAL5	2.49E-07	0.14
	ST3GAL1	5.23E-14	0.22
	ST6GAL1	2.62E-21	0.23
	ST8SIA3	3.49E-23	0.26
Group 3γ-Group 4α	NANS	2.13E-28	-0.42
	ELN	1.58E-03	-0.15
	ST3GAL4	1.63E-06	-0.14
	NEU1	1.94E-04	-0.13
	B4GALNT1	3.50E-08	0.2
	CMAS	5.46E-17	0.35
	ST8SIA2	5.28E-21	0.41
	ST6GAL2	3.10E-40	0.54
	ST8SIA3	2.52E-41	0.6
Group 3γ-Group 4β	NANS	1.54E-32	-0.21
	ELN	1.31E-11	-0.15
	ST3GAL4	1.39E-16	-0.1
	NEU1	8.23E-11	-0.07
	SLC17A5	1.22E-05	-0.05
	ST6GAL1	1.26E-03	-0.03
	ST3GAL3	5.89E-04	-0.02
	ST3GAL6	1.28E-05	0.11
	B4GALNT1	2.72E-07	0.11
	ST3GAL5	1.29E-05	0.14
	ST3GAL1	1.42E-05	0.15
	ST8SIA3	3.22E-15	0.2
Group 3γ - Group 4γ	NANS	1.86E-82	-0.36
	NEU1	2.45E-21	-0.13
	ST3GAL4	3.72E-20	-0.12
	ELN	7.45E-03	-0.05
	SLC17A5	1.40E-04	-0.04
	ST6GALNAC4	6.83E-03	-0.03
	CMAS	1.21E-04	-0.02
	ST3GAL1	3.97E-05	0.15
	ST8SIA3	1.33E-13	0.21
Group 4α - Group 4β	ST6GAL2	9.63E-46	-0.54
	ST8SIA3	7.37E-23	-0.39
	ST8SIA2	4.43E-21	-0.35
	CMAS	7.52E-16	-0.29
	ST6GAL1	3.72E-05	-0.1
	B4GALNT1	5.20E-03	-0.08
	ST3GAL5	4.60E-03	0.16
	NANS	1.20E-06	0.22
Group 4α - Group 4γ	ST6GAL2	6.04E-39	-0.5
	ST8SIA3	1.07E-21	-0.38
	CMAS	1.69E-24	-0.37
	ST8SIA2	1.71E-19	-0.34
	B4GALNT1	2.04E-06	-0.14
Group 4β - Group 4γ	NANS	4.93E-25	-0.15
	CMAS	8.75E-09	-0.08
	ST3GAL6	3.67E-05	-0.07
	B4GALNT1	2.31E-06	-0.06
	NEU1	3.22E-05	-0.06
	ST3GAL5	3.65E-03	-0.04
	CTSA	6.77E-03	-0.04
	ST3GAL3	1.22E-03	0.07
	ELN	2.01E-06	0.1
	ST6GAL1	3.22E-11	0.11

**Figure 5:**
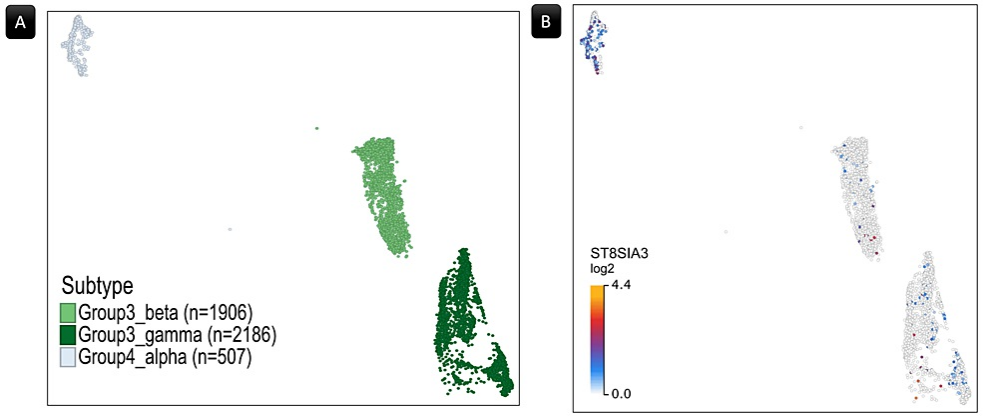
ST8SIA3 gene expression. (A) A UMAP plot depicting the spatial distribution of cells representing Group 4 alpha, Group 3 beta, and gamma medulloblastoma subtypes. Each dot represents a single cell, with distinct clusters representing the three subtypes, highlighting their spatial arrangement. (B) The UMAP plot illustrates the log2-transformed expression levels of the ST8SIA3 gene across the predefined subtypes, revealing a relatively higher expression in Group 4 alpha compared to the Group 3 subtypes.

Furthermore, Group 4α has shown significant expressional differences with Groups 3β and 3γ regarding the expression of another member of the STs, the ST8SIA3 protein (Table 3), also known as the Ganglioside D3 Synthase (GD3S). The ST8SIA3 scored a log2FC of 0.64 with Group 3β (Figure 4H) and 0.60 with 3γ (Figure 4I). Figure 5B depicts the cells with ST8SIA3 log2-transformed expression values in the Group 3β/γ and Group 4α subtypes, highlighting the higher ratio of the cells within the Group 4α cluster.

No other pairwise analyses within the subtypes yielded statistically significant results in terms of gene expression alterations. Table 4 provides a comprehensive summary of the results from the two-group analysis, particularly emphasizing significant expression disparities observed in ST6GAL2 and ST8SIA3. Within the dataset, the WNT subgroup manifests elevated ST6GAL2 expression compared to both Group 3 and Group 4 subgroups and subtypes, except for Group 4α. As depicted in Figure 3C, the cell cluster corresponding to Group 4α demonstrates an ST6GAL2 expression level that is not significantly different from that of the WNT subgroup. Additionally, Group 4α is characterized by a markedly elevated ST8SIA3 expression compared to Group 3β and γ, as illustrated in Figure 5.

**Table 4:** Overview of two-group differences, focusing on the significantly different expression of ST6GAL2 and ST8SIA3 within the sample subgroups and subtypes. The Hodges-Lehmann estimate represents the median difference between paired observations, providing a robust measure of central tendency in non-normally distributed data. The Rank-Biserial Correlation is employed as the effect size for the Mann-Whitney test, indicating the strength and direction of the relationship between groups.

Expression comparison		Statistics	Mean difference	SE	Corrected *P*-value	Hodges-Lehmann estimate	Rank-Biserial correlation
								ST6GAL2							
Subgroups comparison	WNT-Group 3	6.62E+06	1.09	0.04	4.93E-88	3.50E-05	0.44
	WNT-Group 4	7.59E+06	1	0.04	2.27E-75	6.20E-05	0.4
Subtypes comparison	WNT-Group 3α	4.47E+06	1.1	0.04	5.27E-88	4.21E-05	0.45
	WNT-Group 3β	1.00E+06	1.06	0.04	2.63E-63	1.04E-05	0.43
	WNT-Group 3γ	1.15E+06	1.06	0.04	1.94E-66	4.40E-05	0.43
	WNT-Group 4β	4.44E+06	1.05	0.04	8.60E-81	3.49E-05	0.43
	WNT-Group 4γ	2.95E+06	0.99	0.04	2.32E-69	5.48E-05	0.4
ST8SIA3							
Subtypes comparison	Group 3β-Group 4α	2.77E+05	-0.93	0.07	5.48E-48	-47.57	-0.43
	Group 3γ-Group 4α	3.38E+05	-0.89	0.07	2.52E-41	-21.55	-0.39

The inclusion of the MYC gene in our differential analysis alongside the initial set of 38 genes revealed its significant overexpression in Group 3γ compared to the WNT subgroup (log2FC = 0.7, *P* < 0.0001, Figure 4E) and against Group 4α (log2FC = 1.02, *P* < 0.0001, Figure 4I). Within these specific comparative tests, the sole genes displaying significant differential expression were ST6GAL2, exhibiting higher expression in WNT rather than Group 3γ (log2FC = 0.68, *P* < 0.0001, Figure 4E), and ST8SIA3, which was more expressed in Group 4α (log2FC = 0.6, *P* < 0.0001, Figure 4I).

Survival analyses using bulk RNA-seq sample

Following the outcomes of the differential expression analyses, we proceeded with a survival analysis focused on the expression of ST6GAL2 and ST8SIA3 genes.

For ST6GAL2, survival analysis was conducted on samples derived from subsets within the WNT, Group 3, and Group 4 subgroups. Various subset combinations were formed, including those from the subtypes of Groups 3 and 4 (α, β, and γ in each). Nine distinct sample subsets were created for ST6GAL2 analysis, encompassing the WNT with Groups 3 and 4 collectively and each individually, along with each subtype of Groups 3 and 4 (α, β, and γ in each). The Bonferroni-corrected *P*-value threshold for Cox regression and KM log-rank tests was set at 0.006.

**Figure 6:**
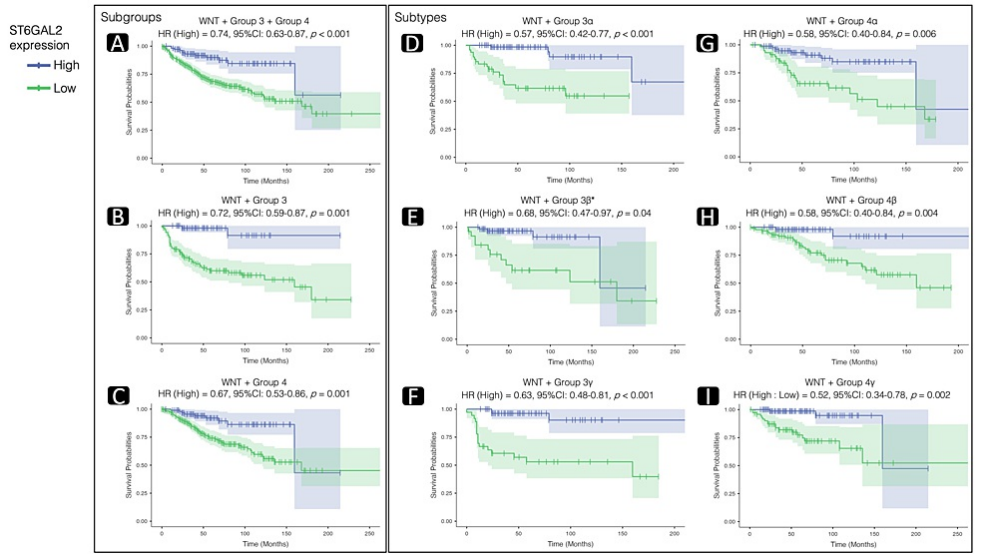
Survival analyses of ST6GAL2 expression. Kaplan-Meier plots illustrate survival probabilities based on ST6GAL2 gene expression. The plots also present the results of Cox regression analysis, including hazard ratios (HR), their 95% confidence interval (CI) range, and *P*-values revealing statistically significant differences (Bonferroni-corrected *P*-value threshold = 0.006). These values demonstrate the hazard ratio (HR) when a one-unit increase in ST6GAL2 occurs. Elevated ST6GAL2 expression correlates with improved overall survival in all sample subsets, except for the subset composed of wingless/integrated (WNT) and Group 3β (E). Further details on log-rank statistics, its *P*-values, the one-, three-, and five-year survival ratios, and subgroup/subtype distribution in the nine subsets can be found in Table 5. (*Cox regression analysis demonstrated no significant result due to its *P*-value greater than the Bonferroni-corrected threshold). ST6GAL2, α-2,6-sialyltransferase 2

All nine subsets exhibited statistically significant differences in overall survival based on stratification by ST6GAL2 gene expression levels (Table 5). Cox regression analyses indicated a notable correlation between elevated ST6GAL2 expression and improved overall survival in eight out of the nine examined sample subsets (Figure 6). Notably, the sole exception was observed in the subset comprising WNT and Group 3β, where although a significant difference in overall survival was identified through the KM log-rank test (χ² = 10.6,* P *= 0.001), the Cox regression analysis did not meet the adjusted significance threshold (HR = 0.68, *P* > 0.006, Figure 6E).

**Table 5:** One-, three-, and five-year survival summary table and the distribution of the subgroups and subtypes in the two strata of Kaplan-Meier survival analysis. The Bonferroni-corrected *P*-value threshold for the multiple comparison analyses regarding ST6GAL2 = 0.006 and ST8SIA3 = 0.025. To view the high and low strata of the ST6GAL2 and ST8SIA3 expressions, refer to Figures 6-7. CI, confidence interval; ST6GAL2, α-2,6-sialyltransferase 2

	One-, three-, and five-year survival						Distribution in the two survival strata		
WNT+Group 3+Group 4 (Log-rank: χ² = 15.4, *P* < 0.0001), illustrated in Figure 6A									
Strata	Time (years)	Number at risk	Number of events	Survival	95% CI		WNT	Group 3	Group 4
					Lower	Upper			
ST6GAL2 = high	1	104	2	98.1%	95.6%	100.0%	94%	9%	14%
	3	74	5	93.0%	88.1%	98.2%			
	5	44	2	89.9%	83.6%	96.6%			
ST6GAL2 = low	1	292	30	90.7%	87.6%	94.0%	6%	91%	86%
	3	204	35	78.6%	74.1%	83.4%			
	5	131	23	68.5%	63.1%	74.4%			
WNT+Group 3 (log-rank: χ² = 20.2, *P* < 0.0001), illustrated in Figure 6B									
Strata	Time (years)	Number at risk	Number of events	Survival	95% CI		WNT	Group 3	
					Lower	Upper			
ST6GAL2 = high	1	55	0	100.0%	100.0%	100.0%	84%	2%	
	3	39	1	98.0%	94.2%	100.0%			
	5	22	0	98.0%	94.2%	100.0%			
ST6GAL2 = low	1	97	24	80.0%	73.1%	87.5%	16%	98%	
	3	65	12	68.4%	60.2%	77.6%			
	5	43	7	59.9%	51.1%	70.3%			
WNT+Group 4 (log-rank: χ² = 11.5, *P* = 0.0007), illustrated in Figure 6C									
Strata	Time (years)	Number at risk	Number of events	Survival	95% CI		WNT	Group 4	
					Lower	Upper			
ST6GAL2 = high	1	95	1	99.0%	96.9%	100.0%	94%	14%	
	3	70	3	95.5%	91.2%	99.9%			
	5	42	2	92.0%	85.9%	98.6%			
ST6GAL2 = low	1	212	7	96.9%	94.6%	99.2%	6%	86%	
	3	150	25	84.4%	79.5%	89.6%			
	5	95	16	74.1%	67.9%	80.9%			
WNT+Group 3α (log-rank: χ² = 20.5, *P* < 0.0001), illustrated in Figure 6D									
Strata	Time (years)	Number at risk	Number of events	Survival	95% CI		WNT	Group 3α	
					Lower	Upper			
ST6GAL2 = high	1	70	0	100.0%	100.0%	100.0%	95%	18%	
	3	50	1	98.4%	95.4%	100.0%			
	5	32	0	98.4%	95.4%	100.0%			
ST6GAL2 = low	1	40	8	83.3%	73.3%	94.6%	5%	82%	
	3	26	6	67.8%	55.0%	83.6%			
	5	16	2	61.6%	48.1%	78.9%			
WNT+Group 3β (log-rank: χ² = 10.6, *P* = 0.001), illustrated in Figure 6E									
Strata	Time (years)	Number at risk	Number of events	Survival	95% CI		WNT	Group 3β	
					Lower	Upper			
ST6GAL2 = high	1	64	0	100.0%	100.0%	100.0%	94%	19%	
	3	44	2	96.7%	92.3%	100.0%			
	5	25	0	96.7%	92.3%	100.0%			
ST6GAL2 = low	1	21	4	84.1%	71.0%	99.7%	6%	81%	
	3	17	2	75.7%	60.5%	94.7%			
	5	11	3	61.5%	44.5%	85.1%			
WNT+Group 3γ (log-rank: χ² = 22, *P* < 0.0001), illustrated in Figure 6F									
Strata	Time (years)	Number at risk	Number of events	Survival	95% CI		WNT	Group 3γ	
					Lower	Upper			
ST6GAL2 = high	1	58	0	100.0%	100.0%	100.0%	88%	6%	
	3	41	2	96.2%	91.2%	100.0%			
	5	23	0	96.2%	91.2%	100.0%			
ST6GAL2 = low	1	25	12	66.7%	52.9%	84.0%	12%	94%	
	3	18	2	60.6%	46.4%	79.1%			
	5	12	2	53.0%	38.2%	73.4%			
WNT+Group 4α (log-rank: χ² = 12.1, *P* = 0.0005), illustrated in Figure 6G									
Strata	Time (years)	Number at risk	Number of events	Survival	95% CI		WNT	Group 4α	
					Lower	Upper			
ST6GAL2 = high	1	80	1	98.8%	96.4%	100.0%	94%	30%	
	3	61	3	94.7%	89.7%	99.9%			
	5	38	2	90.8%	83.8%	98.3%			
ST6GAL2 = low	1	56	1	98.2%	94.9%	100.0%	6%	70%	
	3	38	11	77.3%	66.7%	89.6%			
	5	21	5	65.4%	53.0%	80.5%			
WNT+Group 4β (log-rank: χ² = 9.17, *P* = 0.0025), illustrated in Figure 6H									
Strata	Time (years)	Number at risk	Number of events	Survival	95% CI		WNT	Group 4β	
					Lower	Upper			
ST6GAL2 = high	1	57	0	100.0%	100.0%	100.0%	89%	1%	
	3	41	1	98.1%	94.4%	100.0%			
	5	23	0	98.1%	94.4%	100.0%			
ST6GAL2 = low	1	86	3	96.7%	93.1%	100.0%	11%	99%	
	3	67	5	90.6%	84.6%	97.1%			
	5	40	9	77.1%	67.9%	87.5%			
WNT+Group 4γ (log-rank: χ² = 14.1, *P* = 0.0002), illustrated in Figure 6I									
Strata	Time (years)	Number at risk	Number of events	Survival	95% CI		WNT	Group 4γ	
					Lower	Upper			
ST6GAL2 = high	1	88	0	100.0%	100.0%	100.0%	94%	30%	
	3	59	1	98.7%	96.2%	100.0%			
	5	39	0	98.7%	96.2%	100.0%			
ST6GAL2 = low	1	66	3	95.8%	91.3%	100.0%	6%	70%	
	3	46	9	81.9%	73.1%	91.8%			
	5	30	2	77.3%	67.2%	88.9%			
Group 3β+Group 4α (log-rank: χ² = 2.69, *P* = 0.1), illustrated in Figure 7A									
Strata	Time (years)	Number at risk	Number of events	Survival	95% CI		Group 3β	Group 4α	
					Lower	Upper			
ST8SIA3 = high	1	63	4	94.1%	88.6%	99.9%	30%	78%	
	3	46	12	75.0%	65.1%	86.5%			
	5	27	8	59.7%	48.2%	73.9%			
ST8SIA3 = low	1	32	2	94.3%	86.9%	100.0%	70%	22%	
	3	22	4	82.3%	70.4%	96.2%			
	5	14	2	73.6%	59.1%	91.7%			
Group 3γ+Group 4α (log-rank: χ² = 2.94, *P* = 0.09), illustrated in Figure 7B									
Strata	Time (years)	Number at risk	Number of events	Survival	95% CI		Group 3γ	Group 4α	
					Lower	Upper			
ST8SIA3 = high	1	26	3	89.7%	79.2%	100.0%	10%	34%	
	3	21	3	78.3%	64.2%	95.4%			
	5	12	2	69.2%	53.3%	89.9%			
ST8SIA3 = low	1	67	11	85.8%	78.3%	93.9%	90%	66%	
	3	45	13	68.0%	58.2%	79.5%			
	5	28	7	56.0%	45.3%	69.3%			

**Figure 7:**
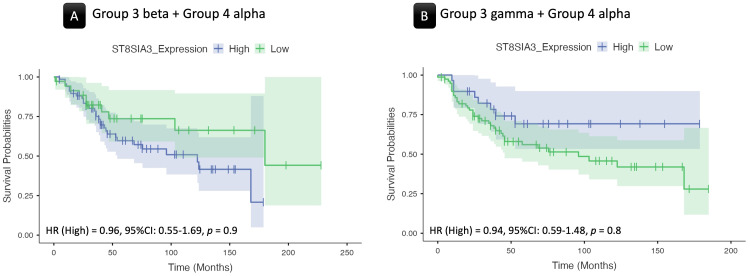
Survival analyses of ST8SIA3 expression. Kaplan-Meier plots illustrate survival probabilities based on ST8SIA3 gene expression. The plots also present the results of Cox regression analysis, including hazard ratios (HRs), their 95% confidence interval (CI) range, and *P*-values revealing statistically significant differences (Bonferroni-corrected *P*-value threshold = 0.025). There was no significant association between ST8SIA3 gene expression and survival.

Within a sample encompassing WNT, Group 3, and Group 4, patients with MB exhibiting high ST6GAL2 expression had a 26% lower mortality risk compared to those with lower expression (Figure 6A). In the subset consisting of WNT and Group 3 (Figure 6B), the reduction was 28%, while in the WNT and Group 4 subset (Figure 6C), it reached 33%. In subsets characterized by the WNT and Group 3 subtypes (Group 3α and Group 3γ), the reduction rate was 43% (Figure 6D) and 37% (Figure 6F), respectively. Similarly, in subsets defined by the WNT and Group 4 subtypes (Group 4α, β, and γ), the reduction rates were 42% (Figure 6G), 42% (Figure 6H), and 48% (Figure 6I), respectively.

Table 5 presents log-rank statistics, *P*-values, and subgroup/subtype distribution in the nine subsets. All analyses demonstrated a significant difference between high and low expression of ST6GAL2 (*P* < 0.006). Notably, the WNT subgroup predominated in the high-expression strata across all tests, while Group 3 and Group 4 subgroups or subtypes (α, β, and γ in each) were more prevalent in the low-expression strata. KM plots (Figure 6) were generated using a relative gene expression cutoff to stratify samples, and Table 5 outlines the one-, three-, and five-year survival ratios for each subset.

For ST8SIA3 expression, survival analysis focused on samples combining Group 4α with either Group 3β or Group 3γ. The Bonferroni-corrected *P*-value threshold was 0.025. In contrast to ST6GAL2, neither the KM log-rank test nor the Cox regression analysis showed a significant association between ST8SIA3 gene expression and survival (*P* > 0.025, Figure 7 and Table 5).

Protein profiling

**Figure 8:**
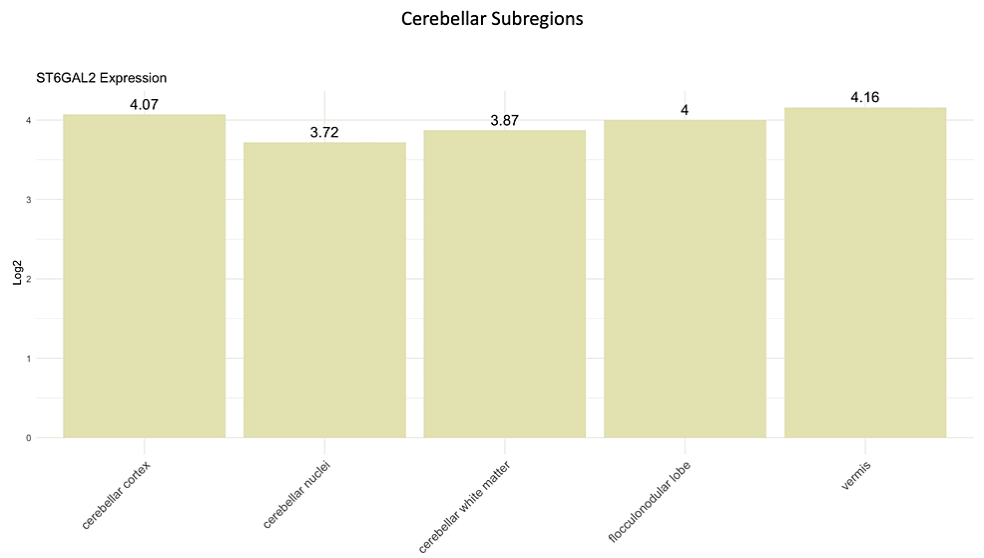
The average log2 of RNA expression of ST6GAL2 in the cerebellar subregions. Data are imported from the Human Protein Atlas (HPA) database (https://www.proteinatlas.org). ST6GAL2, α-2,6-sialyltransferase 2

**Figure 9:**
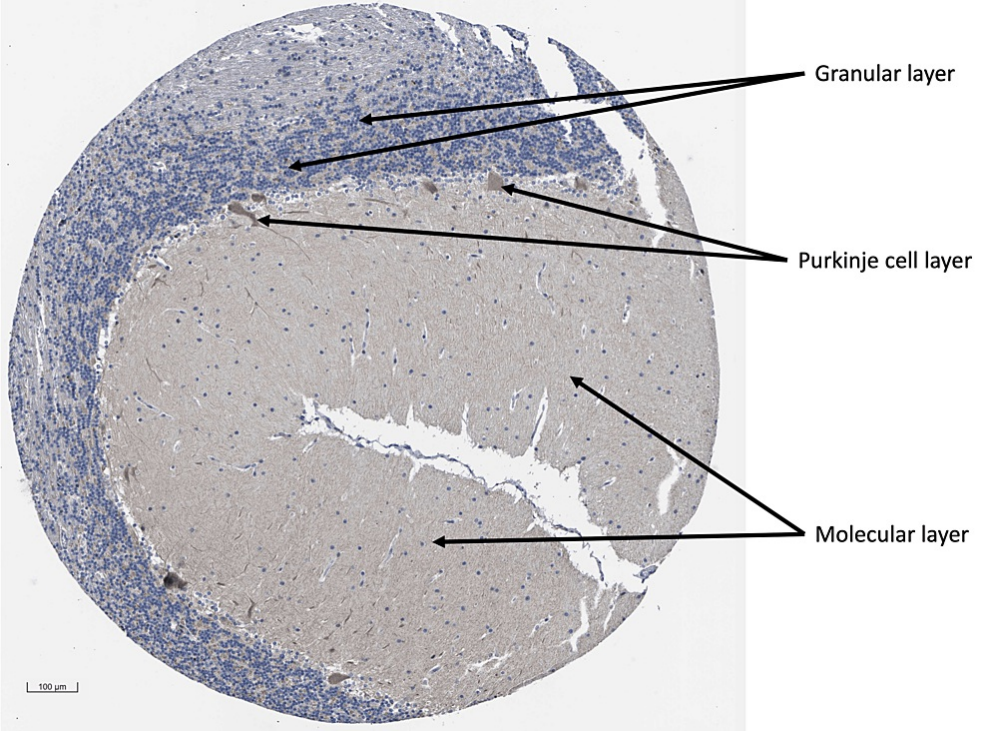
ST6GAL2 immunostaining of the cerebellum. The sample shows medium ST6GAL2 antibody staining in Purkinje cells, low staining in the granular layer, and no detection in the molecular layer. Data are imported from the Human Protein Atlas (HPA) database (https://www.proteinatlas.org). ST6GAL2, α-2,6-sialyltransferase 2

In our pursuit of distinctive gene/protein expression profiles, our primary emphasis was on ST6GAL2, as ST8SIA3 did not present any values in the survival analyses. According to data retrieved from the HPA, ST6GAL2 is expressed in areas relevant to MB origin. The average Log2 of RNA expression of ST6GAL2 in the cerebellum was 4.5 ± 0.84 (Figure 8). Concerning the ST6GAL2 immunostaining of the cerebellum, the HPA indicates medium ST6GAL2 antibody staining in Purkinje cells, low staining in the granular layer, and no detection in the molecular layer (Figure 9).

## Discussion

In the exploration of sialic acid biosynthesis-related gene expression in MB, we conducted a comprehensive analysis. Using the STRING tool, we identified 38 genes associated with sialic acid biosynthesis (Figure 2). Following this, we conducted subsequent differential expression analyses comparing the different MB groups using the scRNA-seq sample, revealing distinct patterns among these 38 genes. Notably, the ST6GAL2 showed the most prominent alterations in the comparisons of WNT against Group 3 and 4 subgroups and their subtypes (Figure 4). Despite variations in cell counts across subgroups, the WNT subgroup exhibited a substantially higher ratio of cells expressing ST6GAL2 (Figure 3C and Table 2).

Building on the differential expression analysis findings, we conducted survival analyses focusing on ST6GAL2 expression. Due to the lack of OS data in the scRNA-seq sample, the survival analyses were made using the bulk tissue RNAseq samples. Our decision to depend on the subgroups and subtypes as criteria for creating sample subsets for the survival analyses is based on the prognostic characteristics of these subgroups and subtypes. The WNT subgroup has a 5-year OS of more than 90%, reaching 100%, while Group 3 is 41.9% to 66.2%, and Group 4 is 66.8% to 82.5% [12,20]. Accordingly, for survival analyses, we created nine distinct sample subsets, combining WNT with Groups 3 and 4 collectively and individually, as well as each subtype of Groups 3 and 4.

The survival analyses revealed a significant correlation between elevated ST6GAL2 expression and reduced mortality rates across all nine subsets (Table 5). Cox regression analyses demonstrated a notable association between elevated ST6GAL2 expression and improved OS in eight out of the nine examined sample subsets (*P* < 0.006, Figure 6). Notably, the sole exception was observed in the subset comprising WNT and Group 3β, where, despite a significant difference in OS identified through the KM log-rank test (*P* = 0.001, Table 5), the Cox regression analysis did not reach the adjusted significance threshold (*P* > 0.006, Figure 6E). This discrepancy suggests a potential lack of robustness in the association within this subset.

Notably, the WNT subgroup, exhibiting the best five-year OS [12,20] and expressing a higher ratio of ST6GAL2 (Figure 3C), consistently dominated the high-expression strata in the KM survival analysis. In contrast, Group 3 and Group 4 subgroups or subtypes were more prevalent in the low-expression strata (Table 5). These findings highlight the potential prognostic significance of ST6GAL2 in MB, particularly within specific molecular subgroups.

Several studies have proven that sialic acid holds prominence in cancer biology. Aberrant sialylation, particularly the addition of sialic acid to glycan chains on cell surfaces, strongly correlates with multiple facets of cancer, including tumor growth, metastasis, immune evasion, and therapy resistance [3,5,21]. Sialic acid fosters tumorigenesis, supporting evasion from apoptosis, facilitating metastasis formation, and fortifying resistance to therapy [3]. The overexpression of STs, the enzymes responsible for sialic acid addition, intensifies tumor metastasis by bolstering immune evasion, promoting invasion and migration, and diminishing chemotherapy efficacy [6,10,21]. Conversely, hyposialylation (decreased sialic acid levels on cell surfaces) also contributes to metastatic spread and chemotherapy evasion in breast cancer.

An example of this multifaceted role is the intricate involvement in breast cancer of α2,6-sialylation explored by Lin et al. and Dashzeveg et al. [22,23]. Both studies delved into the effects of manipulating α2,6-sialylation levels in mammary carcinoma cells, discovering that upregulated sialylation enhances invasion capacity and collagen IV adhesion while diminishing cell-cell adhesion. Conversely, downregulated α2,6-sialylation leads to intensified homotypic cell-cell adhesion and reduced collagen IV adhesion. Dashzeveg et al. concentrated on circulating tumor cells (CTCs) in breast cancer, revealing that CTC clusters resistant to chemotherapy exhibit a specific loss of α-2,6-sialyltransferase 1 (ST6GAL1)-catalyzed α2,6-sialylation. This loss is linked to cellular dormancy, chemotherapy evasion, and increased metastatic seeding. The study identified Podocalyxin-like (PODXL) as a glycoprotein substrate of ST6GAL1, suggesting it as a potential target to counteract chemotherapy-evading metastasis. While Lin et al. scrutinized the direct impact on carcinoma cells, Dashzeveg et al. underscored the dynamics of CTC clusters and their response to therapies in the realm of α2,6-sialylation.

**Figure 10:**
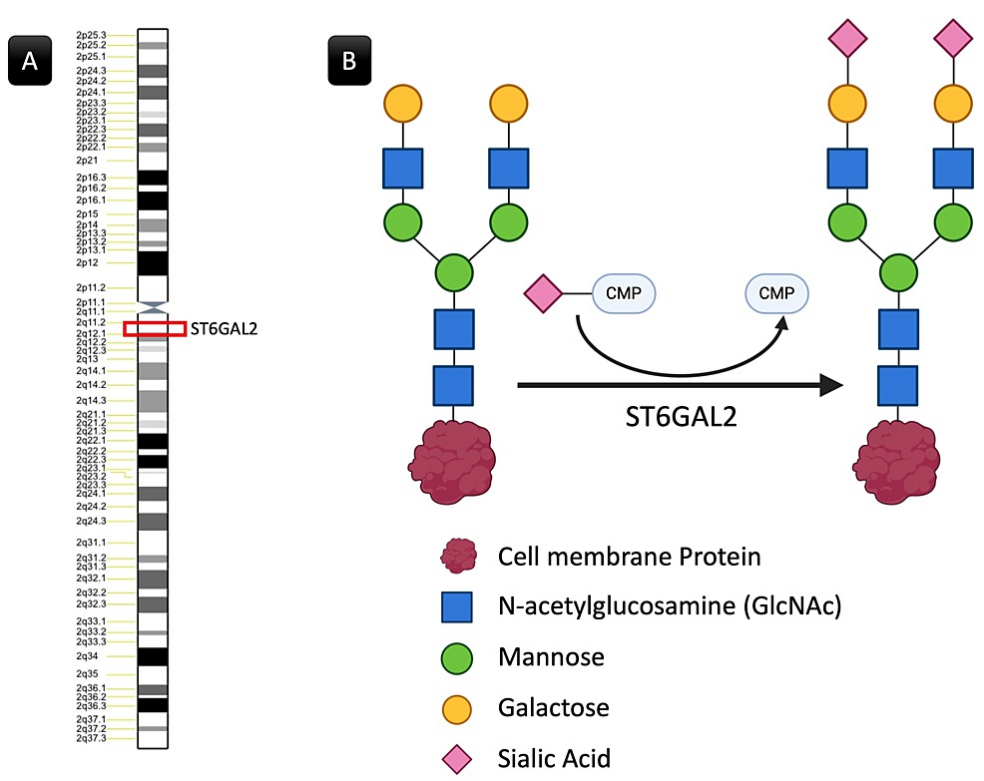
The ST6Gal2 location and function. (A) ST6GAL2 is on chromosome 2 between positions 11.2 and 12.1 on the long arm. (B) ST6GAL2 is involved in sialylation, which is the addition of sialic acid residues to glycoproteins and glycolipids. Specifically, ST6Gal2 catalyzes the transfer of sialic acid to the terminal galactose residue of glycoproteins, forming an alpha-2,6 linkage. Image (B) was constructed on https://www.biorender.com. ST6GAL2, α-2,6-sialyltransferase 2

The ST6GAL2, discovered in 2002, differs from ST6GAL1 in substrate specificity and tissue expression pattern [24,25]. While the ST6GAL1 gene is expressed in almost all human tissues, ST6GAL2 shows a restricted tissue-specific pattern of expression, mostly expressed in fetal and adult brains. The ST6GAL2 gene is located on chromosome 2 (2q11.2-q12.1) (Figure 10). It spans over 85 kb of human genomic DNA, consisting of at least eight exons, and shares a similar genomic structure with the ST6GAL1 gene. Both the ST6GAL1 and ST6GAL2 catalyze the α-2,6-SA of glycoproteins and oligosaccharides [24,26].

In our search in the literature, we found only a single report discussing the role of ST6GAL2 expression in cancers. Liang et al. found that ST6GAL2 upregulation was found to promote tumorigenesis of follicular thyroid carcinoma in vitro and in vivo and promoted tumorigenesis by inactivating the Hippo signaling pathway [27]. The scarcity of data describing the ST6GAL2 role in cancer makes it challenging to hypothesize the cause of the association of the downregulation of this enzyme with poorer OS.

Considering the notably contrasting five-year overall survival rates between the WNT (reaching 100%) and the Group 3γ subtype (41.9%), characterized by MYC amplification [12], we conducted a differential expression analysis encompassing MYC and genes associated with sialic acid biosynthesis. As anticipated, MYC exhibited significant differential expression, being overexpressed in Group 3γ compared to WNT (log2FC = 0.70, *P* < 0.0001). Strikingly, the singular gene besides MYC that displayed noteworthy differential expression in this comparison was ST6GAL2 (log2FC = 0.68, *P* < 0.0001), exhibiting lower expression in Group 3γ while being overexpressed in WNT (Figure 4E). Intriguingly, survival analyses revealed a positive correlation between elevated ST6GAL2 expression and improved overall survival, aligning with mortality risk reductions ranging from 26% to 48%. This suggests that despite MYC amplification and the adverse prognosis associated with Group 3γ, the upregulation of ST6GAL2 may potentially contribute to a more favorable outcome in patients with MB. Further experimental validation is warranted to unravel the intricate molecular mechanisms underpinning these observations and their clinical implications.

The improved survival rates associated with higher ST6GAL2 expression in patients with MB may be attributed to various potential mechanisms in cancer biology. Elevated ST6GAL2 expression could potentially inhibit tumor progression, enhance the anti-tumor immune response, modulate metastatic potential by influencing adhesion properties, and sensitize cells to therapeutic interventions. Additionally, the observed impact on cellular dormancy, similar to findings in breast cancer [23], suggests that ST6GAL2 may play a role in influencing dormancy-related processes, ultimately contributing to better patient survival. However, these hypotheses are speculative and require further experimental validation, emphasizing the need for detailed investigations into the specific molecular and cellular mechanisms underlying the influence of ST6GAL2 on MB progression and patient outcomes.

This study faces several limitations. While the focus on scRNA-seq samples was intended to address tumor heterogeneity, it hindered direct survival analysis due to insufficient data. To mitigate this issue, we integrated Bulk RNA-seq samples, which provided survival data and molecular classification, into the survival analysis. This approach aimed to compensate for the lack of survival information in the scRNA-seq samples, leveraging our understanding of overall survival outcomes across distinct molecular subgroups and subtypes.

Furthermore, this study is primarily reliant on bioinformatic analyses utilizing publicly available datasets. No experimental procedures involving actual patients were conducted as part of this analysis.

## Conclusions

This study delved into sialic acid biosynthesis-related gene expression in MB using scRNA-seq and bulk RNA-seq samples, revealing significant alterations, particularly in ST6GAL2, across molecular subgroups and subtypes. Elevated ST6GAL2 expressions correlated with improved overall survival in various sample subsets, suggesting potential mechanisms such as inhibiting tumor progression and enhancing immune response. However, these hypotheses necessitate experimental validation.
